# (4*RS*)-3-Benzyl 5-methyl 2,6-dimethyl-4-(4-nitro­phen­yl)-1,4-dihydro­pyridine-3,5-dicarboxyl­ate

**DOI:** 10.1107/S1600536809049435

**Published:** 2009-11-25

**Authors:** Bing-zhu Zhang, Yan-ji Wang, Feng-xia Sun, Xu Zhang, Wei Wang

**Affiliations:** a College of Chemical Engineering, Hebei University of Technology, Tianjin 300130, People’s Republic of China; b College of Chemical and Pharmaceutical Engineering, Hebei University of Science and Technology, Shijiazhuang 050018, People’s Republic of China; c Hebei University of Technology, Tianjin 300130, People’s Republic of China

## Abstract

In the title compound, C_23_H_22_N_2_O_6_, the crystal packing is stabilized by inter­molecular N—H⋯O hydrogen bonds, which link the mol­ecules into chains running parallel to the *c* axis. Inter­molecular C—H⋯O hydrogen bonds are also present in the structure.

## Related literature

The title compound is a nefidipine analogue. For the use of nefidipine-type 4-aryl-1,4-dihydro­pyridine-3,5-dicarboxylic diesters in the treatment of cardiovascular disease, see: Goldmann & Stoltefuss (1991 ▶); Yiu & Knaus (1999 ▶). For the structure of 5-ethoxy­carbonyl-2,6-dimethyl-4-(3-nitro­phen­yl)-1,4-dihydro­pyridine-3-carboxylic anhydride ethyl acetate solvate, see: Sun *et al.* (2006 ▶). For hydrogen-bond motifs, see: Etter *et al.* (1990 ▶). 
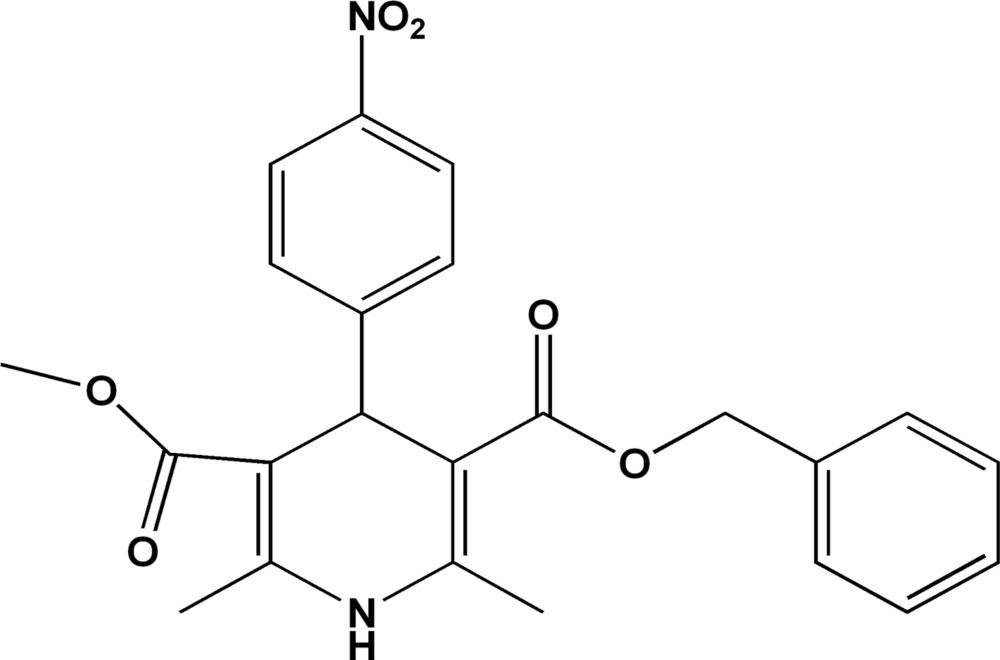


## Experimental

### 

#### Crystal data

C_23_H_22_N_2_O_6_
*M* *_r_* = 422.43
Monoclinic, 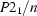

*a* = 9.6527 (19) Å
*b* = 11.043 (2) Å
*c* = 19.883 (4) Å
β = 100.23 (3)°
*V* = 2085.9 (7) Å^3^
*Z* = 4
Mo *K*α radiation
μ = 0.10 mm^−1^
*T* = 293 K
0.26 × 0.20 × 0.10 mm

#### Data collection

Rigaku MM007 diffractometer
Absorption correction: multi-scan (*CrystalClear*; Rigaku, 2005 ▶) *T*_min_ = 0.975, *T*_max_ = 0.990
16526 measured reflections
4745 independent reflections
3087 reflections with *I* > 2σ(*I*)
*R*_int_ = 0.040

#### Refinement

*R*[*F* ^2^ > 2σ(*F* ^2^)] = 0.061
*wR*(*F* ^2^) = 0.188
*S* = 1.02
4745 reflections
288 parameters
H atoms treated by a mixture of independent and constrained refinement
Δρ_max_ = 0.30 e Å^−3^
Δρ_min_ = −0.20 e Å^−3^

### 

Data collection: *CrystalClear* (Rigaku, 2005 ▶); cell refinement: *CrystalClear*; data reduction: *CrystalClear*; program(s) used to solve structure: *SHELXS97* (Sheldrick, 2008 ▶); program(s) used to refine structure: *SHELXL97* (Sheldrick, 2008 ▶); molecular graphics: *SHELXTL* (Sheldrick, 2008 ▶); software used to prepare material for publication: *SHELXTL*.

## Supplementary Material

Crystal structure: contains datablocks global, I. DOI: 10.1107/S1600536809049435/fb2168sup1.cif

Structure factors: contains datablocks I. DOI: 10.1107/S1600536809049435/fb2168Isup2.hkl

Additional supplementary materials:  crystallographic information; 3D view; checkCIF report

## Figures and Tables

**Table 1:** Hydrogen-bond geometry (Å, °)

*D*—H⋯*A*	*D*—H	H⋯*A*	*D*⋯*A*	*D*—H⋯*A*
N1—H1⋯O1^i^	0.89 (2)	2.13 (3)	3.003 (2)	167 (2)
C15—H15⋯O4^ii^	0.93	2.43	3.303 (3)	157
C7—H7*B*⋯O1^i^	0.96	2.55	3.436 (3)	154

Symmetry codes: (i) 
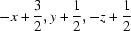
; (ii) 
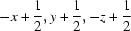
.

**Fig. 1.**
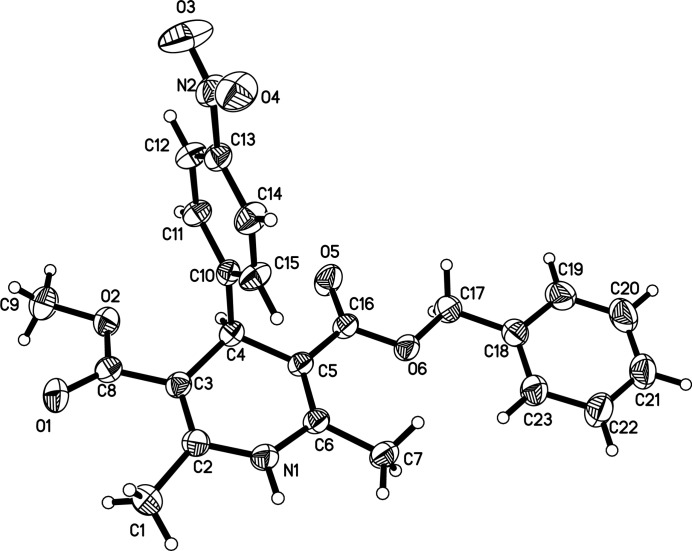
A view of the title molecule. The displacement ellipsoids are drawn at the 30% probability level. The H atoms are represented by spheres of arbitrary radii.

## supplementary crystallographic information

### Comment

4-Aryl-1,4-dihydropyridine-3,5-dicarboxylic diesters of the nefidipine type have become almost indispensable for the treatment of cardiovascular diseases since they first appeared on the market in 1975 (Yiu & Knaus, 1999; Goldmann & Stoltefuss, 1991). The structure of the title compound, 2,6-dimethyl-4-(4-nitro-phenyl)-1,4-dihydro-pyridine-3,5 -dicarboxylic acid 3-benzyl ester 5-methyl ester, is a nefidipine analogue.

Fig. 1 shows the title molecule. In the dihydropyridine ring, the atom C4 is displaced from the mean plane formed by the remaining atoms of the same ring by 0.312 (1) Å. The dihedral angle between the C10//C11//C12//C13//C14//C15 benzene ring and the N1//C2//C3//C5//C6 plane is 89.26 (1)°. This value corresponds well to the structure of 5-ethoxycarbonyl-2,6-dimethyl-4-(3-nitrophenyl)-1,4-dihydropyridine-3- carboxylic anhydride ethyl acetate solvate (Sun & Yu, 2006).

The intermolecular N—H···O hydrogen bonds link the molecules along *c* axis. The graph set is C(7) (Etter *et al.*, 1990).

### Experimental

2,6-Dimethyl-4-(4-nitro-phenyl)-1,4-dihydro-pyridine-3,5-dicarboxylic acid monoethyl ester (332 mg, 1 mmol) and dicyclohexyl-carbodiimide (206 mg, 1 mmol) were dissolved in 28 ml of CH_2_Cl_2_. Phenyl methanol (108 mg, 1 mmol) was added dropwise to the solution at 278 K. The reaction mixture was stirred at 276-279 K for further 9 h. The solvent CH_2_Cl_2_ was removed by evaporation in vacuum at 293 K. The product was purified by chromatography on a silica gel column (eluted by ethyl acetate and petroleum, 1:5) at room temperature. The purified product weighted 350 mg with the yield 83%. Yellow block crystals were obtained by slow evaporation from a solution of ethyl acetate and methanol (1:1) at room temperature.

### Refinement

All the hydrogen atoms could have been discerned in the difference electron density map. Nevertheless, all the H atoms attached to the carbon atoms were constrained in the riding motion approximation. C_aryl_—H=0.93, C_methyl_—H=0.96, C_methylene_—H=0.97, C_methine_—H=0.98 Å while *U*_iso_(H)=1.2*U*_eq_(C_aryl_/_methylene_/_methine_) or 1.5*U*_eq_(C_methyl_). The coordinates as well as the isotropic displacement parameter of the amino hydrogen involved in the N-H···O hydrogen bond were freely refined.

### Crystal data

**Table:**

C_23_H_22_N_2_O_6_	*F*(000) = 888
*M**_r_* = 422.43	*D*_x_ = 1.345 Mg m^−^^3^
Monoclinic, *P*2_1_/*n*	Melting point = 470.0–471.0 K
Hall symbol: -P 2yn	Mo *K*α radiation, λ = 0.71073 Å
*a* = 9.6527 (19) Å	Cell parameters from 4898 reflections
*b* = 11.043 (2) Å	θ = 2.1–27.5°
*c* = 19.883 (4) Å	µ = 0.10 mm^−^^1^
β = 100.23 (3)°	*T* = 293 K
*V* = 2085.9 (7) Å^3^	Block, yellow
*Z* = 4	0.26 × 0.20 × 0.10 mm

### Data collection

**Table:**

Rigaku MM007 diffractometer	4745 independent reflections
Radiation source: rotating anode	3087 reflections with *I* > 2σ(*I*)
confocal	*R*_int_ = 0.040
Detector resolution: 7.31 pixels mm^-1^	θ_max_ = 27.5°, θ_min_ = 2.1°
ω and φ scans	*h* = −12→12
Absorption correction: multi-scan (*CrystalClear*; Rigaku, 2005)	*k* = −10→14
*T*_min_ = 0.975, *T*_max_ = 0.990	*l* = −25→25
16526 measured reflections	

### Refinement

**Table:**

Refinement on *F*^2^	Secondary atom site location: difference Fourier map
Least-squares matrix: full	Hydrogen site location: difference Fourier map
*R*[*F*^2^ > 2σ(*F*^2^)] = 0.061	H atoms treated by a mixture of independent and constrained refinement
*wR*(*F*^2^) = 0.188	*w* = 1/[σ^2^(*F*_o_^2^) + (0.1057*P*)^2^] where *P* = (*F*_o_^2^ + 2*F*_c_^2^)/3
*S* = 1.02	(Δ/σ)_max_ = 0.002
4745 reflections	Δρ_max_ = 0.30 e Å^−^^3^
288 parameters	Δρ_min_ = −0.20 e Å^−^^3^
0 restraints	Extinction correction: *SHELXL97* (Sheldrick, 2008), Fc^*^=kFc[1+0.001xFc^2^λ^3^/sin(2θ)]^-1/4^
81 constraints	Extinction coefficient: 0.045 (6)
Primary atom site location: structure-invariant direct methods	

### Special details

**Table:**

Geometry. All e.s.d.'s (except the e.s.d. in the dihedral angle between two l.s. planes) are estimated using the full covariance matrix. The cell e.s.d.'s are taken into account individually in the estimation of e.s.d.'s in distances, angles and torsion angles; correlations between e.s.d.'s in cell parameters are only used when they are defined by crystal symmetry. An approximate (isotropic) treatment of cell e.s.d.'s is used for estimating e.s.d.'s involving l.s. planes.
Refinement. Refinement of *F*^2^ against ALL reflections. The weighted *R*-factor *wR* and goodness of fit *S* are based on *F*^2^, conventional *R*-factors *R* are based on *F*, with *F* set to zero for negative *F*^2^. The threshold expression of *F*^2^ > σ(*F*^2^) is used only for calculating *R*-factors(gt) *etc*. and is not relevant to the choice of reflections for refinement. *R*-factors based on *F*^2^ are statistically about twice as large as those based on *F*, and *R*- factors based on ALL data will be even larger.

### Fractional atomic coordinates and isotropic or equivalent isotropic displacement parameters (Å^2^)

**Table:**

	*x*	*y*	*z*	*U*_iso_*/*U*_eq_	
O1	0.89299 (15)	0.58542 (14)	0.20521 (9)	0.0717 (5)	
O2	0.78376 (14)	0.54481 (13)	0.09912 (8)	0.0653 (5)	
O3	0.2026 (3)	0.23247 (18)	0.15616 (13)	0.1172 (8)	
O4	0.14892 (19)	0.35571 (16)	0.23061 (10)	0.0839 (5)	
O5	0.38588 (17)	0.74335 (14)	−0.02349 (8)	0.0739 (5)	
O6	0.31955 (14)	0.92631 (12)	0.00562 (7)	0.0554 (4)	
N1	0.61176 (17)	0.88992 (16)	0.19025 (9)	0.0534 (5)	
N2	0.21172 (19)	0.33054 (17)	0.18454 (11)	0.0637 (5)	
C1	0.8265 (2)	0.8162 (2)	0.25856 (12)	0.0705 (7)	
H1A	0.8042	0.7733	0.2973	0.106*	
H1B	0.8365	0.9009	0.2691	0.106*	
H1C	0.9130	0.7857	0.2478	0.106*	
C2	0.70961 (19)	0.79849 (18)	0.19804 (10)	0.0500 (5)	
C3	0.69608 (18)	0.70649 (16)	0.15252 (9)	0.0456 (5)	
C4	0.56533 (18)	0.69660 (15)	0.09720 (9)	0.0435 (4)	
H4	0.5945	0.6705	0.0547	0.052*	
C5	0.49105 (18)	0.81767 (15)	0.08450 (9)	0.0444 (4)	
C6	0.51034 (19)	0.90593 (16)	0.13245 (10)	0.0463 (5)	
C7	0.4354 (2)	1.02481 (18)	0.13165 (12)	0.0607 (6)	
H7A	0.4610	1.0756	0.0966	0.091*	
H7B	0.4616	1.0637	0.1752	0.091*	
H7C	0.3356	1.0114	0.1226	0.091*	
C8	0.8009 (2)	0.60980 (17)	0.15669 (11)	0.0524 (5)	
C9	0.8818 (3)	0.4482 (2)	0.09446 (17)	0.0889 (9)	
H9A	0.9751	0.4808	0.0986	0.133*	
H9B	0.8566	0.4083	0.0511	0.133*	
H9C	0.8790	0.3910	0.1306	0.133*	
C10	0.46877 (17)	0.59917 (15)	0.11870 (9)	0.0421 (4)	
C11	0.4607 (2)	0.48421 (17)	0.09037 (10)	0.0503 (5)	
H11	0.5129	0.4664	0.0565	0.060*	
C12	0.3770 (2)	0.39576 (17)	0.11132 (11)	0.0542 (5)	
H12	0.3721	0.3189	0.0919	0.065*	
C13	0.30103 (19)	0.42372 (17)	0.16151 (10)	0.0493 (5)	
C14	0.3074 (2)	0.53621 (18)	0.19149 (12)	0.0591 (6)	
H14	0.2558	0.5530	0.2257	0.071*	
C15	0.3917 (2)	0.62377 (17)	0.16985 (11)	0.0555 (5)	
H15	0.3969	0.7002	0.1898	0.067*	
C16	0.3957 (2)	0.82418 (16)	0.01799 (10)	0.0486 (5)	
C17	0.2198 (2)	0.92855 (19)	−0.05783 (11)	0.0602 (6)	
H17A	0.1611	0.8567	−0.0612	0.072*	
H17B	0.2699	0.9284	−0.0960	0.072*	
C18	0.1296 (2)	1.03911 (19)	−0.06098 (11)	0.0567 (5)	
C19	0.0127 (2)	1.0456 (2)	−0.11286 (14)	0.0782 (7)	
H19	−0.0074	0.9821	−0.1438	0.094*	
C20	−0.0742 (3)	1.1467 (3)	−0.11858 (17)	0.0891 (9)	
H20	−0.1521	1.1502	−0.1536	0.107*	
C21	−0.0473 (2)	1.2403 (2)	−0.07396 (15)	0.0808 (8)	
H21	−0.1062	1.3076	−0.0783	0.097*	
C22	0.0669 (3)	1.2349 (2)	−0.02266 (14)	0.0790 (7)	
H22	0.0855	1.2983	0.0084	0.095*	
C23	0.1556 (2)	1.1348 (2)	−0.01659 (12)	0.0680 (6)	
H23	0.2339	1.1327	0.0182	0.082*	
H1	0.622 (2)	0.952 (2)	0.2186 (12)	0.068 (7)*	

### Atomic displacement parameters (Å^2^)

**Table:**

	*U*^11^	*U*^22^	*U*^33^	*U*^12^	*U*^13^	*U*^23^
O1	0.0723 (9)	0.0628 (10)	0.0728 (11)	0.0165 (8)	−0.0062 (8)	0.0123 (8)
O2	0.0582 (8)	0.0574 (9)	0.0780 (11)	0.0130 (7)	0.0060 (7)	−0.0124 (8)
O3	0.160 (2)	0.0591 (12)	0.151 (2)	−0.0422 (12)	0.0787 (17)	−0.0187 (12)
O4	0.0931 (12)	0.0766 (12)	0.0923 (14)	−0.0070 (9)	0.0443 (11)	0.0172 (9)
O5	0.0962 (11)	0.0602 (10)	0.0566 (9)	0.0213 (9)	−0.0106 (8)	−0.0152 (7)
O6	0.0631 (8)	0.0441 (8)	0.0538 (8)	0.0061 (6)	−0.0039 (6)	−0.0001 (6)
N1	0.0600 (10)	0.0466 (10)	0.0499 (10)	0.0026 (8)	−0.0004 (8)	−0.0076 (8)
N2	0.0698 (11)	0.0484 (11)	0.0754 (13)	−0.0024 (9)	0.0195 (10)	0.0131 (9)
C1	0.0691 (13)	0.0687 (15)	0.0658 (14)	0.0023 (12)	−0.0100 (11)	−0.0061 (12)
C2	0.0527 (10)	0.0487 (11)	0.0476 (11)	−0.0023 (9)	0.0059 (8)	0.0020 (8)
C3	0.0485 (10)	0.0413 (10)	0.0474 (10)	−0.0005 (8)	0.0093 (8)	0.0040 (8)
C4	0.0501 (10)	0.0389 (9)	0.0424 (10)	0.0019 (8)	0.0103 (8)	0.0010 (8)
C5	0.0496 (10)	0.0372 (9)	0.0460 (10)	−0.0009 (8)	0.0074 (8)	0.0013 (8)
C6	0.0526 (10)	0.0382 (9)	0.0478 (10)	−0.0007 (8)	0.0079 (8)	0.0000 (8)
C7	0.0724 (13)	0.0434 (11)	0.0625 (13)	0.0050 (9)	0.0020 (10)	−0.0086 (9)
C8	0.0541 (11)	0.0433 (11)	0.0591 (12)	−0.0026 (9)	0.0084 (9)	0.0058 (9)
C9	0.0723 (15)	0.0665 (16)	0.126 (3)	0.0215 (13)	0.0130 (16)	−0.0199 (16)
C10	0.0465 (9)	0.0378 (9)	0.0414 (9)	0.0051 (8)	0.0059 (7)	0.0014 (7)
C11	0.0637 (12)	0.0423 (10)	0.0468 (11)	−0.0006 (9)	0.0147 (9)	−0.0060 (8)
C12	0.0700 (12)	0.0377 (10)	0.0563 (12)	−0.0033 (9)	0.0148 (10)	−0.0064 (9)
C13	0.0518 (10)	0.0421 (10)	0.0544 (11)	−0.0004 (8)	0.0106 (9)	0.0082 (8)
C14	0.0694 (13)	0.0478 (11)	0.0670 (14)	0.0027 (10)	0.0311 (11)	−0.0010 (10)
C15	0.0709 (12)	0.0385 (10)	0.0622 (13)	−0.0008 (9)	0.0255 (10)	−0.0089 (9)
C16	0.0574 (11)	0.0388 (10)	0.0490 (11)	0.0010 (9)	0.0079 (9)	−0.0004 (8)
C17	0.0665 (12)	0.0573 (13)	0.0514 (12)	0.0026 (10)	−0.0045 (10)	0.0010 (10)
C18	0.0542 (11)	0.0551 (12)	0.0588 (12)	−0.0015 (9)	0.0048 (9)	0.0132 (10)
C19	0.0733 (15)	0.0717 (16)	0.0805 (17)	−0.0048 (13)	−0.0107 (13)	0.0083 (13)
C20	0.0638 (14)	0.0880 (19)	0.105 (2)	0.0088 (14)	−0.0128 (14)	0.0223 (17)
C21	0.0667 (15)	0.0744 (17)	0.099 (2)	0.0178 (13)	0.0083 (14)	0.0191 (15)
C22	0.0870 (17)	0.0657 (15)	0.0821 (18)	0.0202 (14)	0.0090 (14)	0.0014 (13)
C23	0.0690 (13)	0.0630 (14)	0.0666 (15)	0.0106 (11)	−0.0021 (11)	0.0033 (11)

### Geometric parameters (Å, °)

**Table:**

O1—C8	1.220 (2)	C9—H9A	0.9600
O2—C8	1.336 (3)	C9—H9B	0.9600
O2—C9	1.440 (2)	C9—H9C	0.9600
O3—N2	1.217 (3)	C10—C11	1.385 (2)
O4—N2	1.217 (3)	C10—C15	1.390 (3)
O5—C16	1.208 (2)	C11—C12	1.378 (3)
O6—C16	1.345 (2)	C11—H11	0.9300
O6—C17	1.445 (2)	C12—C13	1.375 (3)
N1—C2	1.372 (3)	C12—H12	0.9300
N1—C6	1.382 (2)	C13—C14	1.375 (3)
N1—H1	0.89 (2)	C14—C15	1.381 (3)
N2—C13	1.467 (3)	C14—H14	0.9300
C1—C2	1.509 (3)	C15—H15	0.9300
C1—H1A	0.9600	C17—C18	1.495 (3)
C1—H1B	0.9600	C17—H17A	0.9700
C1—H1C	0.9600	C17—H17B	0.9700
C2—C3	1.352 (3)	C18—C23	1.371 (3)
C3—C8	1.463 (3)	C18—C19	1.389 (3)
C3—C4	1.524 (2)	C19—C20	1.389 (4)
C4—C5	1.517 (2)	C19—H19	0.9300
C4—C10	1.533 (2)	C20—C21	1.357 (4)
C4—H4	0.9800	C20—H20	0.9300
C5—C6	1.353 (3)	C21—C22	1.364 (4)
C5—C16	1.473 (3)	C21—H21	0.9300
C6—C7	1.498 (3)	C22—C23	1.391 (3)
C7—H7A	0.9600	C22—H22	0.9300
C7—H7B	0.9600	C23—H23	0.9300
C7—H7C	0.9600		
			
C8—O2—C9	118.08 (18)	H9B—C9—H9C	109.5
C16—O6—C17	115.58 (14)	C11—C10—C15	118.56 (17)
C2—N1—C6	124.02 (17)	C11—C10—C4	121.51 (17)
C2—N1—H1	120.4 (14)	C15—C10—C4	119.87 (16)
C6—N1—H1	114.2 (14)	C12—C11—C10	121.33 (18)
O4—N2—O3	123.2 (2)	C12—C11—H11	119.3
O4—N2—C13	118.27 (19)	C10—C11—H11	119.3
O3—N2—C13	118.5 (2)	C13—C12—C11	118.50 (18)
C2—C1—H1A	109.5	C13—C12—H12	120.7
C2—C1—H1B	109.5	C11—C12—H12	120.7
H1A—C1—H1B	109.5	C14—C13—C12	121.97 (18)
C2—C1—H1C	109.5	C14—C13—N2	118.81 (19)
H1A—C1—H1C	109.5	C12—C13—N2	119.21 (18)
H1B—C1—H1C	109.5	C13—C14—C15	118.75 (19)
C3—C2—N1	119.65 (17)	C13—C14—H14	120.6
C3—C2—C1	126.96 (18)	C15—C14—H14	120.6
N1—C2—C1	113.36 (17)	C14—C15—C10	120.87 (18)
C2—C3—C8	121.81 (17)	C14—C15—H15	119.6
C2—C3—C4	120.43 (16)	C10—C15—H15	119.6
C8—C3—C4	117.66 (16)	O5—C16—O6	121.41 (17)
C5—C4—C3	111.31 (14)	O5—C16—C5	122.53 (17)
C5—C4—C10	111.85 (14)	O6—C16—C5	116.06 (15)
C3—C4—C10	108.40 (14)	O6—C17—C18	110.09 (17)
C5—C4—H4	108.4	O6—C17—H17A	109.6
C3—C4—H4	108.4	C18—C17—H17A	109.6
C10—C4—H4	108.4	O6—C17—H17B	109.6
C6—C5—C16	125.68 (16)	C18—C17—H17B	109.6
C6—C5—C4	121.06 (16)	H17A—C17—H17B	108.2
C16—C5—C4	113.21 (15)	C23—C18—C19	118.0 (2)
C5—C6—N1	118.83 (16)	C23—C18—C17	124.32 (18)
C5—C6—C7	128.20 (17)	C19—C18—C17	117.6 (2)
N1—C6—C7	112.97 (16)	C20—C19—C18	120.1 (2)
C6—C7—H7A	109.5	C20—C19—H19	120.0
C6—C7—H7B	109.5	C18—C19—H19	120.0
H7A—C7—H7B	109.5	C21—C20—C19	121.1 (2)
C6—C7—H7C	109.5	C21—C20—H20	119.5
H7A—C7—H7C	109.5	C19—C20—H20	119.5
H7B—C7—H7C	109.5	C20—C21—C22	119.4 (2)
O1—C8—O2	121.37 (18)	C20—C21—H21	120.3
O1—C8—C3	127.2 (2)	C22—C21—H21	120.3
O2—C8—C3	111.38 (16)	C21—C22—C23	120.2 (2)
O2—C9—H9A	109.5	C21—C22—H22	119.9
O2—C9—H9B	109.5	C23—C22—H22	119.9
H9A—C9—H9B	109.5	C18—C23—C22	121.2 (2)
O2—C9—H9C	109.5	C18—C23—H23	119.4
H9A—C9—H9C	109.5	C22—C23—H23	119.4
			
C6—N1—C2—C3	−10.8 (3)	C4—C10—C11—C12	178.01 (16)
C6—N1—C2—C1	167.68 (19)	C10—C11—C12—C13	−0.2 (3)
N1—C2—C3—C8	177.12 (17)	C11—C12—C13—C14	−0.6 (3)
C1—C2—C3—C8	−1.2 (3)	C11—C12—C13—N2	−179.83 (17)
N1—C2—C3—C4	−6.5 (3)	O4—N2—C13—C14	−1.7 (3)
C1—C2—C3—C4	175.23 (19)	O3—N2—C13—C14	177.4 (2)
C2—C3—C4—C5	21.3 (2)	O4—N2—C13—C12	177.56 (19)
C8—C3—C4—C5	−162.18 (16)	O3—N2—C13—C12	−3.3 (3)
C2—C3—C4—C10	−102.15 (19)	C12—C13—C14—C15	0.7 (3)
C8—C3—C4—C10	74.4 (2)	N2—C13—C14—C15	179.89 (18)
C3—C4—C5—C6	−21.9 (2)	C13—C14—C15—C10	0.1 (3)
C10—C4—C5—C6	99.5 (2)	C11—C10—C15—C14	−0.8 (3)
C3—C4—C5—C16	160.48 (16)	C4—C10—C15—C14	−178.00 (17)
C10—C4—C5—C16	−78.09 (19)	C17—O6—C16—O5	4.1 (3)
C16—C5—C6—N1	−175.08 (17)	C17—O6—C16—C5	−176.17 (17)
C4—C5—C6—N1	7.6 (3)	C6—C5—C16—O5	178.0 (2)
C16—C5—C6—C7	4.1 (3)	C4—C5—C16—O5	−4.5 (3)
C4—C5—C6—C7	−173.18 (18)	C6—C5—C16—O6	−1.8 (3)
C2—N1—C6—C5	10.3 (3)	C4—C5—C16—O6	175.70 (16)
C2—N1—C6—C7	−169.03 (19)	C16—O6—C17—C18	172.55 (17)
C9—O2—C8—O1	−3.0 (3)	O6—C17—C18—C23	11.5 (3)
C9—O2—C8—C3	178.18 (18)	O6—C17—C18—C19	−169.27 (19)
C2—C3—C8—O1	15.2 (3)	C23—C18—C19—C20	−0.2 (4)
C4—C3—C8—O1	−161.27 (19)	C17—C18—C19—C20	−179.5 (2)
C2—C3—C8—O2	−166.02 (18)	C18—C19—C20—C21	−0.2 (4)
C4—C3—C8—O2	17.5 (2)	C19—C20—C21—C22	−0.1 (4)
C5—C4—C10—C11	134.51 (18)	C20—C21—C22—C23	0.6 (4)
C3—C4—C10—C11	−102.39 (19)	C19—C18—C23—C22	0.7 (4)
C5—C4—C10—C15	−48.4 (2)	C17—C18—C23—C22	180.0 (2)
C3—C4—C10—C15	74.7 (2)	C21—C22—C23—C18	−1.0 (4)
C15—C10—C11—C12	0.9 (3)		

### Hydrogen-bond geometry (Å, °)

**Table:**

*D*—H···*A*	*D*—H	H···*A*	*D*···*A*	*D*—H···*A*
N1—H1···O1^i^	0.89 (2)	2.13 (3)	3.003 (2)	167 (2)
C15—H15···O4^ii^	0.93	2.43	3.303 (3)	157
C7—H7B···O1^i^	0.96	2.55	3.436 (3)	154

Symmetry codes: (i) −*x*+3/2, *y*+1/2, −*z*+1/2; (ii) −*x*+1/2, *y*+1/2, −*z*+1/2.
